# The effectiveness of completely and incompletely sealed first permanent molars on caries prevention

**DOI:** 10.1002/cre2.280

**Published:** 2020-02-14

**Authors:** Lenka Likar Ostrc, Jana Suklan, Alenka Pavlič

**Affiliations:** ^1^ Department of Paediatric and Preventive Dentistry, Faculty of Medicine University of Ljubljana Ljubljana Slovenia; ^2^ Institute of Cellular Medicine Newcastle University Newcastle upon Tyne UK; ^3^ NIHR Newcastle in vitro Diagnostics Co‐operative Newcastle upon Tyne UK; ^4^ Unit of Paediatric and Preventive Dentistry University Medical Centre Ljubljana Ljubljana Slovenia

**Keywords:** child, dental caries, dental fissures, preventive medicine

## Abstract

Fissure sealants are effective caries preventive measure. However, a dilemma has been expressed more than once, whether incompletely sealed fissures provides sufficient protection against caries. Dental examinations were performed in 88 children, aged 8 and then 4 years later at 12 years. All first permanent molars (FPMs), as diagnosed at the age of 8, were divided into three groups: nonsealed, incompletely and completely sealed. Four years later caries incidence and changes in presence and quality of fissure sealant were analyzed. At the age of 8 and 12 mean DMFT were 0.73 ± 1.24 and 3.48 ± 3.04, respectively. 71.59% of the 8‐year‐olds and 78.41% of the 12‐year‐olds had at least one sealed FPM. At the age of 8, 154 FPMs were completely sealed and 42 FPMs were incompletely sealed. Four years later, 81.17%, 71.43% and 69.4% of FPMs were healthy (sound or with noncavitated caries) in the baseline groups completely sealed, incompletely sealed and nonsealed FPMs, respectively. Incompletely sealed fissures were more susceptible to caries development than completely sealed fissures. It is important that incompletely sealed fissures are resealed as soon as possible.

## 1 INTRODUCTION

Over the last decades, there has been a decline in dental caries prevalence among children and adolescents. Nevertheless, dental caries is still a major health problem worldwide (Ahovuo‐Saloranta, Forss, Hiiri, Nordblad, & Makela, 2016), affecting as many as 60–90% of children (WHO, 2017). While some decrease in caries prevalence has been observed, the number of caries lesions developed on occlusal surfaces remains high (Hiiri, Ahovuo‐Saloranta, Nordblad, & Makela, 2006), the lower the caries incidence in the population, the higher the proportion of occlusal caries (Batchelor & Sheiham, 2004). The occlusal surfaces of both the first permanent molars (FPMs) and the second permanent molars (SPMs) have the greatest risk of caries development (Demirci, Tuncer, & Yuceokur, 2010). Although the occlusal surface represents only 15% of all dental surfaces, as much as 88% of all caries lesions develops in pits and fissures of FPMs and SPMs (Rethman, 2000). In 18‐year‐olds, almost half of all surfaces affected by caries are on the occlusal surface (Norrisgaard, Qvist, & Ekstrand, 2016).

Occlusal caries start in pits and fissures, which are particularly susceptible for caries development due to their complex morphology, that prevents both natural cleaning by saliva and mechanical cleaning by toothbrush (Bromo, Guida, Santoro, Peciarolo, & Eramo, 2011; Disney et al., 1992). The highest probability of occlusal caries development is during the time of a tooth's eruption (Carvalho, 2014). Dental plaque accumulates more abundantly on the occlusal surfaces of not fully erupted molars, compared to molars with proper occlusal contact (Carvalho, 2014). Additionally, post‐eruptive mineralization of the outer enamel of a not fully erupted tooth is not completed (Kataoka, Sakuma, Wang, Yoshihara, & Miyazaki, 2007).

In children and adolescents, fissure sealants are suggested to be effective and safe in preventing the development of dental caries (Ahovuo‐Saloranta, Forss, Walsh, et al., 2013), sealed fissure systems effectively prevent caries development or slows down and even stops further progression of initial caries lesion (Wright et al., 2016a). The purpose of fissure sealing is to protect the FPM as soon as it erupts. Fissures, tightly sealed with a resin‐based sealer, make a physical barrier that prevents nutrients entering the fissures. It limits the growth and metabolism of microorganisms present in the fissure, therefore excretion of acids is reduced (Ahovuo‐Saloranta et al., 2016). With fluorides added, as for example, in GIC sealers, fluorides contribute to the remineralisation of initial caries lesions as they are released from the sealing material (Zhou et al., 2012).

Effectiveness of pits and fissures sealing in preventing dental caries has often been reported. Two systematic reviews, which pool data on the effectiveness of fissure sealing from 38 studies and from 23 studies (Wright et al., 2016b), found strong evidence that supports the benefits of sealing permanent molars in children and adolescents. Results of the first above mentioned systematic review showed that resin‐based sealants reduced caries by between 11% and 51% compared to nonsealed teeth, reevaluated after 24 months (Ahovuo‐Saloranta et al., 2017). Results from the second above‐mentioned systematic review showed a 76% risk reduction in the development of new carious lesions in participants who received sealants compared with those whose teeth were not sealed, after 2–3 year follow up (Wright, Tampi, et al., 2016b). After a 7 year follow‐up, children and adolescents with sealed teeth had a caries incidence of 29%, whereas in those without sealed dental fissures caries incidence was 74% (Wright, Tampi, et al., 2016b). A protective effect of pit and fissure sealants, particularly for children and adolescents at high caries risk, is further confirmed by meta‐analysis of 19 studies (15 RCT and 4 systematic reviews) (Neusser, Krauth, Hussein, & Bitzer, 2014).

A dilemma has been expressed more than once regarding the fissure sealing; whether in incompletely (partially) sealed fissures the risk of caries development is increased. Yet, publication on the risk for caries development in partly sealed fissures is scarce. Some studies conclude that sealant loss is not associated with risk of caries (Muller‐Bolla, Courson, Lupi‐Pegurier, et al., 2018; Simonsen, 1991). Analysis of the results of seven studies concludes that teeth, in which the sealing was partially or completely lost, did not have an increased risk of caries development compared to unsealed teeth. The authors conclude that fissure sealants are indicated even if there is no possibility for a regular check‐up (Griffin, Gray, Malvitz, & Gooch, 2009). On the other hand, it has been reported that incompletely sealed fissure systems present an increased risk for caries development compared to completely sealed teeth, which indicates that sealants should be regularly monitored and reapplied whenever necessary, to be effective (Chestnutt, Schafer, Jacobson, & Stephen, 1994; Ismail & Gagnon, 1995; Veiga, Ferreira, Correia, & Preira, 2014). Generally, it is accepted that all sealed tooth surfaces should be monitored regularly; whenever found defective, sealers should be reapplied in order to maintain the marginal integrity (Welbury, Raadal, & Lygidakis, 2004).

The aim of this study was to determine caries morbidity as well as the extent and quality of fissure sealing placement on FPMs in the group of children, aged 8 and then at the age of 12. Furthermore, we evaluated what was the protective effect of fissure sealing against dental caries over the 4‐year period; especially regarding the quality of placed sealing.

## 2 MATERIAL AND METHODS

### 2.1 Study population

Hundred and twenty‐three school children, 58 boys and 65 girls, all 8 years old were invited to the study. Children included in the study had no systemic disease, neither motor nor cognitive impairment. Prior to any examination, written informed consent was obtained from all participants and their parents. The study was approved by the Slovenian National Committee for Medical Ethics (No. 0120–34/2017/4).

**Figure 1:**
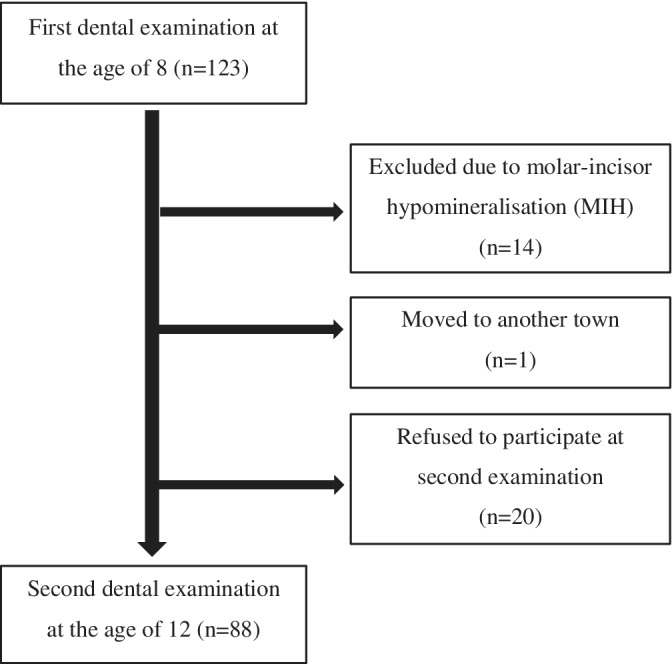
The flowchart of participants included in the study

The first examination was performed on 123 children. Of these, 14 participants were subsequently excluded due to molar‐incisor hypomineralization (MIH). Four years later, 109 children were recalled. Of those, 88 of the then 12‐year‐old children (37 boys and 51 girls) responded positively to the invitation and were included in the study (Figure 1).

### 2.2 Examination procedures

The first dental examination was performed by three dentists who were calibrated. Four years later, the second dental examination of the same group of children was performed by one of the dentist. The other two dentists, who were not interested in further participation, agreed that the study would be carried out in the form and with the co‐workers as presented. Before the first dental examination, all dentists had an ICDAS‐II calibration course, evaluating the condition of tooth surfaces and the presence of caries on 20 photographs according to the International Caries Detection and Assessment System (ICDAS‐II) (International Caries Detection and Assessment System (ICDAS) Coordinating Committee, 2017). The evaluations were repeated after 1 month. Intraexaminer and interexaminer reproducibility was determined with/by intraclass correlation coefficient (ICC) and kappa coefficient, respectively. The ICC for intraexaminer reproducibility showed perfect agreement regarding both surface condition assessment (ICC = 0.97 on first, and 1.00 on second evaluation) and caries presence (ICC = 0.95 on first, and 0.96 on second evaluation). Interexaminer reproducibility also showed excellent to perfect agreement regarding surface condition assessment (weighted kappa 0.86–1.00) and caries presence (weighted kappa 0.64–0.94).

All dental examinations were done in a dental office on a dental chair, under artificial light, using dental mirrors and blunt probe. Before starting with the dental examination, the dentist performed professional tooth cleaning with a professional dental brush and toothpaste. After the cleaning procedure, all the dental surfaces were initially examined wet. Then, the surfaces were dried with air for 5 s and checked again.

Signs of dental caries were recorded in accordance with ICDAS, and later on these results were converted to DMFT (number of decayed‐D, missing‐M and filled‐F permanent teeth) and dmft (number of decayed‐d, missing‐m, filled‐f primary teeth). For cavitated caries presence, ICDAS codes 3–6 were recorded as decayed‐D in DMFT, and ICDAS codes 0–2 as “healthy” (i.e., sound or noncavitated caries lesion). If a tooth was filled and decayed, it was recorded as decayed. At the first examination, missing primary canines and molars were recorded as lost due to caries. If a primary tooth and its permanent successor were present, only the permanent tooth was taken into consideration.

At the baseline, in 8‐year‐olds, each FPM's occlusal surface was evaluated in respect of the presence of signs of caries and/or dental treatment (i.e., sealing or filling). Given the average eruption time of FPMs, these teeth could have been sealed at any time during the previous 2 years. All teeth were sealed with a resin‐based sealer, and sealings had been placed by different dentists. After excluding the filled FPMs, FPMs were divided into three groups in relation to the presence of sealing: (a) nonsealed (ICDAS codes 00, 01 and 02), (b) not completely sealed (ICDAS codes 10, 11 and 12) and (c) completely sealed FPMs (ICDAS codes 20, 21 and 22). Four years later (in 12‐year‐olds), all FPMs were reevaluated.

### 2.3 Data analysis

The statistical analysis was conducted with IBM SPSS 23.0 and the *p* value was set at .05 or higher. Friedman's nonparametric test was used to detect differences in occurrence of caries at the age of 8 and at the age of 12 on the occlusal surfaces of FPMs in comparison to other surfaces. Furthermore, we used Wilcoxon's signed‐rank test to check for differences in the number of cavitated carious lesions on individual surfaces of FPMs in 8‐ and 12‐year‐olds. The Pearson chi‐square test was used to detect statistically significant associations between the two examinations (at the baseline and the second one); to determine a possible association in the clinical status of the FPMs (sound, noncavitated or cavitated caries lesion), the presence of fillings on FPMs, and the number and the quality (incompletely or completely sealed) of FPMs' sealing. A two way analysis of variance was used to test the difference between the two examinations according to the quality of the fissure sealing at age 8 and caries at age 12. For each of the three groups of FPMs (nonsealed, incompletely or completely sealed FPMs), proportions of healthy teeth, teeth with noncavitated and cavitated caries lesions, filled or extracted teeth were calculated. Based on the results of the first and the second examination, relative risk and odds ratio of deterioration due to caries in each of the three groups were calculated.

## 3 RESULTS

Caries morbidity in the examined group of children was high (Table 1). The number of “caries free” children (DMFT = 0) at the first and the second dental examination changed significantly (43.022, *p* = .000). Similarly, a statistically significant difference was obtained when comparing the number of children with permanent teeth not affected by caries (D*MFT = 0) at the first and the second examination (43.022, *p* = .000).

**Table 1:** Number and proportion of children with teeth not affected by caries lesions at the first (8‐year‐olds) and the second (12‐year‐olds) examination

	8‐year‐olds						12‐year‐olds					
	Total		Boys		Girls		Total		Boys		Girls	
	*n*	%	*n*	%	*n*	%	*n*	%	*n*	%	*n*	%
DMFT = 0	59	67.05	28	75.68	31	60.78	14	15.91	5	13.51	9	17.65
D*MFT = 0	26	29.55	12	32.43	14	27.45	1	1.14	0	0.00	1	1.96
Dmft = 0	10	11.36	4	10.81	6	11.76	‐	‐	‐	‐	‐	‐
DMFT+DMFT = 0	8	9.09	4	10.81	4	7.84	‐	‐	‐	‐	‐	‐

At the ages of 8 and 12, participants had on average 12.31 ± 2.04 and 26.61 ± 2.55 permanent teeth erupted, respectively. Over the 4‐year period, increase in the mean DMFT and especially in the mean D*MFT was obvious (Table 2).

**Table 2:** Mean DMFT, D*MFT and dmft of the group of children assessed at the first (8‐year‐olds) and the second (12‐year‐olds) examination

	8‐year‐olds						12‐year‐olds					
	Total		Boys		Girls		Total		Boys		Girls	
	Mean	*SD*	Mean	*SD*	Mean	*SD*	Mean	*SD*	Mean	*SD*	Mean	*SD*
DMFT	0.73	1.24	0.54	1.23	0.86	1.24	3.48	3.04	3.27	3.03	3.63	3.02
D*MFT	1.70	1.64	1.70	1.63	1.71	1.63	11.75	4.45	11.27	4.57	12.10	4.55
Dmft	5.26	3.51	5.24	3.52	5.27	3.51	—	—	—	—	—	—

From the whole group of 88 participants, 64 and 306 permanent teeth were included to DMFT at the age of 8 and 12, respectively. Of these, 62 teeth (96.88% of all permanent teeth included in DMFT of 8‐years olds) and 148 teeth (48.37% of all permanent teeth included in DMFT of 12‐years olds) were FPMs. At the age of eight, 26 FPMs (7.39% of all FPMs) were identified with cavitated caries lesion and 36 FPMs (10.23% of all FPMs) with fillings. Four years later, 58 FPMs (16.48% of all FPMs) had cavitated caries lesion, 88 FPMs (25.00% of all FPMs) were filled and two FPMs were extracted (0.57% of all FPMs).

**Figure 2:**
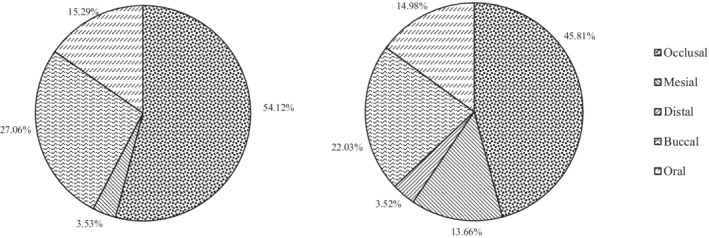
Proportion of FPM's surfaces affected with cavitated caries lesions (untreated cavitated or filled) (a) at the first (8‐year‐olds) (b) and the second dental examination (12‐year‐olds)

Among individual surfaces of the FPMs, the occlusal surface was the most often affected by cavitated caries (in the form of cavitated caries lesions or fillings) both at the first and at the second dental examinations (see Figure 2). At the age of 8 and 12, there were 46 and 104 FPMs occlusal surfaces affected due to cavitated caries, respectively. In the 8‐year‐olds, a statistically significant difference was found on the occlusal surface compared to the mesial, distal, buccal and oral surfaces. In the same way, a statistically significant difference was found in the 12‐year‐olds regarding the numbers of filled or cavitated caries lesions on occlusal FPM's surface compared to any other tooth surface (Table 3). Furthermore, we detected a statistically significant increase in FPMs surfaces affected by cavitated caries. The results showed that the increase of caries on the occlusal surface was 2.29 times higher in the second examination compared to the first one, the mesial surface experienced a major increase in average FPMs surfaces affected by cavitated caries (untreated cavitated and filled) by 12.3 times, the increase in caries on the buccal surface was 2.23 fold, while the increase in caries on the oral surface was 2.67 times the one that was measured at the first examination.

**Table 3:** Number of first permanent molars' (FPMs) surfaces affected by cavitated caries (untreated cavitated and filled)

	8‐year‐olds				12‐year‐olds				
Surface	M (*SD*)	Min	Max	Wilcoxon's signed rank test (sig.)	M (*SD*)	Min	Max	Wilcoxon's signed rank test (sig.)	Friedman's test (sig.)
Occlusal	0.52 (1.08)	0.00	4.00		1.19 (1.29)	0.00	4.00		0.000
Mesial	0.03 (0.18)	0.00	1.00	0.000	0.37 (0.67)	0.00	3.00	0.000	0.000
Distal	0 (0)	0.00	0.00	0.000	0.10 (0.40)	0.00	2.00	0.000	0.005
Buccal	0.26 (0.62)	0.00	2.00	0.021	0.58 (0.87)	0.00	4.00	0.000	0.086
Oral	0.15 (0.47)	0.00	2.00	0.000	0.40 (0.74)	0.00	3.00	0.000	0.000

*Note*: M = mean; SD = standard deviation; Min = minimum value; Max = maximum value; Wilcoxon's signed rank test was used to compare FPMs affected by cavitated caries between occlusal and any other surface, while Friedman's test was used to compare FPMs affected by cavitated caries at the base line and at the second examination.

Of all sound FPMs at the baseline (nonsealed or sealed), only 28.3% of the FPMs remained sound 4 years later, while 55% developed noncavitated and 5.6% cavitated caries lesions (Table 4). In the groups of sound, noncavitated and cavitated FPMs, as diagnosed at the age of 8, 11.2%, 29.1% and 100% of FPMs were filled, respectively 4 years later. The association between the clinical status of the tooth at the baseline (sound, noncavitated or cavitated caries lesion) and the presence of a filling at the second examination was statistically significant (*χ*^2^=173.284, df = 9, *p* > .05).

**Table 4:** The development of new cavitated defects on occlusal surfaces of first permanent molars (FPMs) from first (8‐year‐olds) to the second (12‐year‐olds) examination

			12‐year‐olds			
			Sound	Noncavitated caries	Cavitated caries	Filled or extracted
	Total	352	75	173	14	90
8‐year‐olds	Sound	251	71	138	14	28
	Noncavitated caries	55	4	35	0	16
	Cavitated caries	24	0	0	0	24
	Filled	22	0	0	0	22

The number of sealed FPMs in the examined group of children was high. At the first examination, 71.59% of 8‐year‐old children had at least one FPM sealed (Table 5). At the second examination, 78.41% of all children had at least one FPM sealed. The distribution of placement of fissure sealants between the four groups of FPMs (upper/lower, left/right) was comparable (Table 6). We tested whether statistically significant differences were present within each observational point and between the observations. A two way ANOVA, which included the FPMs distribution of fissure sealant placement and both observation measurements, showed that none of the covariates have a direct effect on all sealed FPMs or on completely sealed FPMs. While the number of sealed FPMs at the baseline and at the second examination was not much different, the quality of FPM's sealing dropped dramatically between the two observational points. There are two interactions that provide a statistically significant effect: between sealed FPMs and the distribution of fissure sealant placement (F = 9.744, *p* < .05), as well as between sealed FPMs and the two observations (987.977, *p* < .001).

**Table 5:** Sealed FPMs in the examined group of children

	8‐year‐olds			12‐year‐olds		
	Total	Boys	Girls	Total	Boys	Girls
	n	n	n	n	n	n
No. of children with at least one sealed FPM	63	26	37	69	29	40
Mean number of sealed FPMs per child	2.23	2.22	2.24	2.30	2.30	2.29

**Table 6:** Distribution of fissure sealant placement, in‐between upper right, upper left, lower left and lower right FPMs

	8‐year‐olds		12‐year‐olds	
	All sealed FPMs	Completely sealed FPMs only	All sealed FPMs	Completely sealed FPMs only
FPM	n	n	n	n
16	51	43	53	15
26	46	36	46	9
36	48	38	51	10
46	51	37	52	7
Total	196	154	202	41

Of all sealed FPMs in 8‐year‐olds (*n* = 196) and 12‐year‐olds (*n* = 202), only 78.57% and 20.3% of FPMs were completely sealed, respectively. Not only were many of the FPMs not sealed appropriately at the age of eight, but reapplication of dental sealers was very poor throughout the period of 4 years (Table 7).

**Table 7:** Number of nonsealed, incompletely sealed and completely sealed FPMs, as observed at the second examination, in the three groups (nonsealed, incompletely sealed and completely sealed FPMs) determined at the first examination

			12‐year‐olds			
			Nonsealed	Incompletely sealed	Completely sealed	Filled or extracted
8‐year‐olds	Total	330	60	161	41	68
	Nonsealed	134	46	37	14	37
	Incompletely sealed	42	10	18	2	12
	Completely sealed	154	4	106	25	19

At the baseline, 55.68% of all FPMs were (incompletely or completely) sealed. During the 4‐year‐period, an additional 51 teeth were sealed in the group of nonsealed FPMs; 27.45% of those teeth were sealed completely and 72.55% insufficiently. At the age of 12, the majority of FPMs from all three of the baseline established groups (nonsealed, incompletely and completely sealed) had placed incomplete sealing. Table 7 shows that only 16.23% of all completely sealed FPMs at age 8 remained completely sealed at age 12, while the vast majority of teeth from this group were incompletely sealed (68.83%) 4 years later. On the other hand, it could also be noted that 10.44% of FPMs that were not sealed at age 8 were completely sealed by the age of 12 and 27.61% of FPMs that were not sealed at age 8 were partially sealed by the age of 12. The association between the two observations is statistically significant according to Pearson chi‐square statistics (χ^2^ = 9.087, df = 2, *p* < .05).

In order to determine the caries preventive effect of presence and quality of fissure sealing, we divided FPMs, as observed in the 8‐year olds, into one of the three groups: (a) nonsealed, (b) incompletely sealed and (c) completely sealed. Four years later, the clinical condition of each of the FPMs was reevaluated. At the age of 12, only one‐fifth of all FPMs (21.3%) were sound, while 49.14% had signs of noncavitated caries lesions (Table 8). In the groups of nonsealed, incompletely and completely sealed FPMs only 23.88%, 14.28% and 24.03% of FPMs, respectively remained sound after the 4‐year period. Approximately half of the FPMs from each of the three groups developed signs of noncavitated caries over the 4‐year period. At the baseline, 6.25% of all FPMs were already filled. Four years later, 25% of all FPMs were filled and 0.56% extracted. At the age of 12, 30.6%, 28.57%, and 18.83% FPMs were affected due to cavitated caries (untreated cavitated leasons or filled FPMs) in the group of nonsealed, incompletely and completely sealed FPMs, respectively. The association in proportions of cavitated caries lesions between the two observations was statistically significant (*χ*^2^=84.239, df = 9, *p* < .001).

**Table 8:** Clinical findings on FPMs, present due to caries (noncavitated or cavitated caries lesions, filled or extracted first permanent molars ‐ FPMs) as observed at the second examination, in a group of nonsealed, incompletely or completely sealed FPMs at the baseline

			12‐year‐olds			
			Sound	Noncavitated caries	Cavitated caries	Filled or extracted
8‐year‐olds	Total	352	75	173	14	90
	Nonsealed	134	32	61	4	37
	Incompletely sealed	42	6	24	0	12
	Completely sealed	154	37	88	10	19
	Filled	22	0	0	0	22

**Figure 3:**
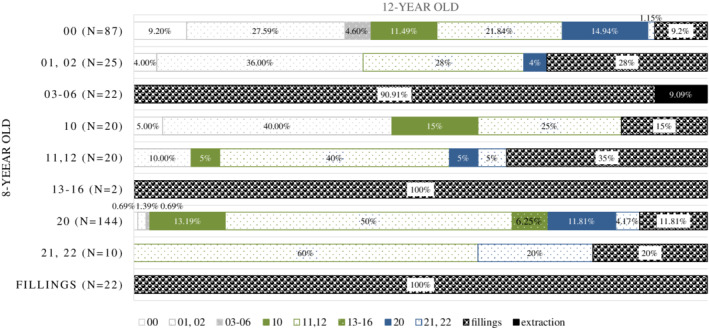
Clinical descriptions of each the FPM's occlusal surface in relation to the presence of caries and/or fissure sealing as observed at the first examination at the age of 8 (vertically on the left) and the outcome after the four‐year‐period (various changes are listed with proportions in horizontal bars). Legend: 00: Sound; 01, 02: Noncavitated caries; 03–06: Cavitated caries; 10: Sound and incompletely sealed; 11, 12: Noncavitated caries and incompletely sealed; 13–16: Cavitated caries and incompletely sealed; 20: Sound and completely sealed; 21, 22: Noncavitated caries and completely sealed; F: Filled; E: Extracted

Figure 3 presents summarized findings from the first and the second dental examination for each of the FPMs in respect of the presence and characteristics of caries as well as dental procedures (i.e., sealing or filling placement). The data in the first three rows presents at the baseline nonsealed FPMs, in the fourth to sixth, incompletely sealed, in the seventh and eight rows, completely sealed, and in the ninth row, filled FPMs. Of all at the baseline nonsealed FPMs (*n* = 134), almost a third were incompletely sealed (37 FPMs) 4‐years later; 27 of those also had noncavitated caries. Additionally, in this group 4 FPMs developed cavitated caries, 7 FPMs were filled and two FPMs were extracted during this period of time. In the group of incompletely sealed FPMs at the baseline (*n* = 42) sealing was reapplied only on two FPMs. In the group of incompletely sealed FPMs, 24 had signs of noncavitated caries and 12 were filled at the second examination. Of all at the baseline completely sealed FPMs (*n* = 154), there were 89 incompletely sealed FPMs (80 with signs of noncavitated caries and 9 with cavitated caries), and 19 FPMs were filled 4‐years later.

**Figure 4:**
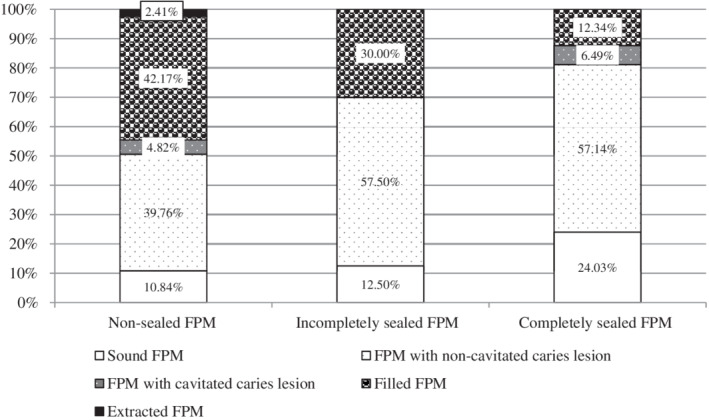
In the first, second, and third columns are shown the proportions of the clinical outcomes (sound, noncavitated caries, cavitated caries, filled or extracted tooth), as found in the 12‐year‐olds, in the three groups of FPMs: nonsealed, incompletely sealed and completely sealed FPMs as determined at the age of eight

Further, for every FPM from each of the three distinct groups (nonsealed, incompletely or completely sealed FPMs) progression of caries lesion and quality of sealing or placement of filling during those 4‐years was established. For this purpose data was omitted on teeth which had been sealed or resealed during the 4‐year period. Results showed that the proportion of degradation differed significantly between the three groups. In the 12‐year‐olds, the highest proportion of filled FPMs was found in the group of at the baseline nonsealed FPMs (Figure 4). Moreover, two FPMs in this group had already been extracted. The highest proportion of sound FPMs in 12 year‐old children was found in the group of at the baseline completely sealed FPMs (24.03%), while the values were half in the group of incompletely sealed FPMs (12.5%) and the group of nonsealed FPMs at the baseline (10.84%).

During the 4‐year period, caries development and/or its progression was noticed within 62.5% of FPMs. Caries development from sound to noncavitated surface and from sound to cavitated caries lesion were found in 39.2% and 3.98% of FPMs, respectively. Decline from any condition to a placement of a filling or tooth extraction was observed in 18.75% and 0.57% of FPMs, respectively. Irreversible conditions (i.e., caries cavitation, dental fillings and extractions) was determined as deterioration of the tooth. At the second examination, almost one quarter of all FPMs underwent a caries process which resulted in an irreversible clinical outcome (i.e., lesion progression into untreated caries cavitation, need for placement of dental filling or even need for a FPM to be extracted due to caries).

As a group, completely sealed FPMs was compared to nonsealed and to incompletely sealed FPMs, estimated relative risk of deterioration was 0.605 and 0.715, respectively. The probability of deterioration for nonsealed FPMs, incompletely and completely sealed FPMs was 0.441, 0.357, and 0.232, respectively. The estimated odds ratio for a relative risk of deterioration of nonsealed FPMs compared to completely sealed FPMs was 1.948, while incompletely sealed FPMs compared to completely sealed FPMs was 1.539.

## 4 DISCUSSION

According to this study, efficiency of fissure sealing in caries prevention is related to the quality of sealed fissure. Between the three groups of FPMs (nonsealed, incompletely and completely sealed FPMs) a significant difference in 4‐year caries incidence was found. At the second examination proportions of FPM, affected by cavitated caries (untreated caries, filled or extracted due to caries), were 30.6%, 28.57% and 18.83% for the group of at the baseline nonsealed, incompletely and completely sealed FPMs, respectively. Chestnutt et al. (1994) report similar results; after a period of 4 years reported proportions of FPMs, affected by cavitated caries (untreated, filled or extracted), are 21.4%, 22.9% and 14.4% for a group of nonsealed, incompletely and completely sealed FPMs, respectively. On the contrary, Simonsen (1991) states that sealants are safe and effective even 15 years after single application and Muller‐Bolla et al. (2018) claim that clinical condition 2 years after a loss of fissure sealant is not associated with a higher risk of caries. Compared to the result obtained in this study, with 83.8% of partially retained or completely lost sealant after the 4 year period, reported sealant retention in the study of Simonsen (1991) (82% after 5 years, 56.7% after 10 years and 27.6% after 15 years) and Muller‐Bolla et al. (2018) (70.4% after 2 years) are higher. In a systematic review, comprising of five studies analyzing fissure‐sealing efficacy over a period of 4 years, it is stated that the risk for caries development in incompletely sealed fissures is similar to nonsealed fissure systems (Griffin et al., 2009). Authors also conclude that the inability of a child to participate further in a retention‐check‐up examination should not disqualify a child from receiving dental sealants. However, the study does not identify what is the preventive effect of incompletely sealed fissures in comparison to completely sealed fissures. In contrast, in the presented study it seemed likely that other factors decisively influenced dental caries development; e.g., poor oral hygiene and/or cariogenic diet. We would argue that in patients with higher risk for caries partially retained sealants pose an additional risk for caries development. Therefore, resealing of incompletely sealed fissures seems to be essential in patients with higher risk for caries development.

As observed in this study, the estimated odds ratio for a relative risk of deterioration over the 4‐year time‐period (i.e., change to caries cavitation, dental filling or tooth extraction) was slightly higher in the group of nonsealed FPMs (1.948) than in the group of incompletely sealed FPMs (1.539) compared to completely sealed FPMs. The difference in the probability of deterioration of completely sealed FPMs, compared to the other two groups, was evident. Results indicated that incompletely sealed FPMs did not protect the fissure system efficiently. Indeed, a clear dissimilarity in the protective effect between incompletely and completely sealed FPMs was identified. The protective effect of incompletely sealed FPMs was significantly reduced compared to completely sealed FPMs.

In this study, the number of sealed FPMs was high. On average, each child had 2.23 and 2.26 sealed FPMs at the first and second examination, respectively. The high proportion of sealed teeth was probably related to the fact that those children had high caries risk, which is an indication for dental sealing placement (Neusser et al., 2014; Welbury et al., 2004). In the examined group of children, high caries morbidity was already present in primary dentition. One of the risk factors for caries development in permanent dentition is the presence of nontreated cavitated caries in primary dentition (Skeie, Raadal, Strand, & Espelid, 2006). In mixed dentition, dental caries of primary molars may influence development of carious lesion on FPMs (Vanderas, Kavvadia, & Papagiannoulis, 2004).

The distribution of fissure sealants placement between the upper (right and left) and lower (right and left) FPMs was comparable. In addition, when proportions of completely sealed FPMs or proportions of FPMs with observed caries deterioration were analysed, no statistically significant difference was found between the upper and the lower FPMs. Contrarily, Lygidakis, Oulis, & Christodoulidis (1994) reported better retention rate on the lower molars compared to the upper. They clarify that on the upper molars only a thin layer of material remains due to the tendency of the material to flow. In this study, of all completely sealed FPMs at the baseline only 16.23% remained completely sealed after a 4‐year period; with similar proportions for the upper and the lower FPMs. Given the high proportion of incompletely sealed upper and lower FPMs in this study, we assumed that poor sealing quality of the lower teeth could be due to premature application of resin‐based sealant on erupting teeth, without use of a rubber‐dam, resulting in saliva contamination and compromised sealant retention.

At the age of 8, as many as one third of the participating children had permanent teeth affected with cavitated caries. Four years later, 85% of the subjects had permanent teeth affected with cavitated caries, with the mean DMFT 3.51. These results differed significantly from previously published results for 12‐year olds in Slovenia, with 64% of the examined adolescents having permanent teeth affected with cavitated caries and mean DMFT of 1.89 (Vrbic & Vrbic, 2016). Reported caries morbidity among 12‐year‐olds in other European countries is also much lower; e.g., in Germany, Norway, Iceland and Portugal caries incidence varies between 31–59.8%, with the mean DMFT between 0.72–1.96 (Agustsdottir et al., 2010; Calado, Ferreira, Nogueira, & Melo, 2017; Haugejorden & Magne Birkeland, 2006; Pieper, Lange, Jablonski‐Momeni, & Schulte, 2013). The discrepancy between the results of this study and the above‐mentioned could be related to the selection as well as to the number of included participants. In the present study, children from three randomly selected Slovenian cities were included, 88 children in total. In the above‐mentioned studies from other European countries from 5–100% of 12‐year‐old populations were included (Agustsdottir et al., 2010; Calado et al., 2017; Haugejorden & Magne Birkeland, 2006; Pieper et al., 2013) and, in the previously published Slovenian study, 1.5% of 12‐year‐olds (Vrbic & Vrbic, 2016).

During the 4‐year follow‐up period, the numbers of caries lesions increased on all FPM's tooth surfaces. Despite the large proportion of sealed FPMs, the occlusal surface was highly affected by caries. Application of fissure sealant is a safe and effective preventive measure against development of a fissure caries. Yet, fissure sealing alone could only prevent caries development to some extent. Additionally, results of this study showed that recommendations on regular follow up of sealed teeth and sealer reapplication if sealed inadequately, was implemented insufficiently (Welbury et al., 2004).

A comparison between the three groups of FPMs (nonsealed, incompletely and completely sealed FPMs) obviously indicates a higher risk of caries in incompletely sealed FPMs compared to completely sealed FPMs. Nevertheless, this comparison has certain shortcomings. In particular, no visual inspection was possible of under at the baseline sealed surfaces. Application of fissure sealing is indicated also on fissure systems with signs of noncavitated caries lesion (Naaman, El‐Housseiny, & Alamoudi, 2017; Wright, Crall, et al., 2016a), therefore it is possible that some of the completely sealed FPMs had signs of caries before sealing was placed. Further, one cannot state with certainty that in the group of the completely sealed FPMs none of these teeth had been incompletely sealed for a certain period of time and then resealed before the second examination. Also, it is probable that some of at the baseline incompletely sealed FPMs were resealed during the 4‐year period, yet again resulting in incomplete sealing at the second examination.

After 4 years, in the examined group of children an especially profound increase was observed in noncavitated caries lesions. Under appropriate conditions, noncavitated caries lesions would remineralize; synchronized implementation of a series of appropriate preventive measures would effectively prevent further caries development. Significance of professional caries prevention measures is widely acknowledged and often exposed; the essential role of simultaneously preventive actions, undertaking by an individual, is every so often less highlighted. In order to reduce high morbidity, implementation of a variety of preventive measures should be incorporated. Professional and individual preventive measures clearly need to be strengthened simultaneously. Some countries report good results when a specially designed national programme is achieved; e.g., Scotland and Portugal (Calado et al., 2017; Macpherson, Ball, Brewster, et al., 2010). In Slovenia, population based caries preventive measures started with the implementation of fluoride tablets in 1956, and was followed by the implementation of topical application of fluorides and fissure sealants in 1970 and in 1980, respectively. As a result, caries prevalence among children was reduced (Vrbic & Vrbic, 2016). Although educational activities (e.g., promotion of individual good oral hygiene and noncariogenic diet) have also been applied, caries preventive action is likely to still be inadequate, and additional information about the importance of one's own role for maintaining good oral health is needed. Children and their parents need to be further educated and motivated about their own contribution in preventing caries and improving their own oral health. Further, it is necessary to identify which specific preventive activities need to be specifically reinforced at the national level, to gain a significant positive effect on improving the oral health of children.

In conclusion, caries morbidity in the examined group of children was high. At the same time, the children had a large proportion of sealed FPMs, predominantly inadequately sealed. The results of this study showed that incompletely sealed fissure systems were more susceptible to the development of caries than completely sealed fissure systems of the FPMs. In particular, in children with a higher caries risk, regular retention‐check‐up of sealed teeth is particularly important; and, whenever identified as incompletely sealed, such a tooth must be resealed.

### 4.1 Why this paper is important to paediatric dentists

- Paediatric dentists are encouraged to implement a regular check‐up of applied pit and fissure sealants and to reseal them, when found incomplete or lost.
- Application of fissure sealants is safe and effective however simultaneous emphasis on implementation of other preventive measures is also required, especially in a population with high caries prevalence.

## AUTHOR CONTRIBUTIONS

L.L.O collected data and wrote the manuscript; J.S. contributed to design of the study, carried out the statistical analysis and wrote the manuscript; A.P. conceived the idea of the study, conducted and wrote the manuscript, and was guide for the whole study.
